# PET-CT in brain disorders: The South African context

**DOI:** 10.4102/sajr.v25i1.2201

**Published:** 2021-11-10

**Authors:** Alexander G.G. Doruyter, Jeannette Parkes, Jonathan Carr, James M. Warwick

**Affiliations:** 1 NuMeRI Node for Infection Imaging, Central Analytical Facilities, Stellenbosch University, Cape Town, South Africa; 2 Division of Nuclear Medicine, Faculty of Medicine and Health Sciences, Stellenbosch University, Cape Town, South Africa; 3 Division of Radiation Oncology, Faculty of Health Sciences, University of Cape Town, Cape Town, South Africa; 4 Division of Neurology, Faculty of Medicine and Health Sciences, Stellenbosch University, Cape Town, South Africa

**Keywords:** brain, positron emission tomography, F-18 fluorodeoxyglucose, F-18 fluoro-dihydroxyphenylalanine, dementia, brain tumour, Parkinson’s disease

## Abstract

Positron emission tomography combined with X-ray computed tomography (PET-CT) has an established role in the management of brain disorders, but may be underutilised in South Africa. Possible barriers to access include the limited number of PET-CT facilities and the lack of contemporary guidelines for the use of brain PET-CT in South Africa. The current review aims to highlight the evidence-based usage of brain Positron emission tomography (PET) in dementia, movement disorders, brain tumours, epilepsy, neuropsychiatric lupus, immune-mediated encephalitides, and brain infections. While being areas of research, there is currently no clinical role for the use of PET-CT in traumatic brain injury or in psychiatric or neurodevelopmental disorders. Strategies to expand the appropriate use of PET-CT in brain disorders are discussed in this article.

## Introduction

Neurological diseases have a high worldwide and local prevalence. In 2015, the age-standardised rate of disability-adjusted life years attributable to neurological conditions in South Africa was estimated at 3549 (95% confidence interval [CI]: 3176–3953) per 100 000.^1^

When neuroimaging is indicated in neurological conditions, this is typically initiated with anatomical techniques such as CT and/or MRI, with or without contrast. In most clinical circumstances, nuclear imaging techniques such as positron emission tomography combined with conventional X-ray computed tomography (PET-CT) are reserved for cases in which the diagnosis remains in doubt after inconclusive anatomical imaging. The strengths and limitations of anatomical and functional techniques (including PET-CT) have previously been reviewed.^2^

PET-CT is now a mature imaging modality in clinical medicine, and has been available in South Africa for more than 15 years. After the injection of a radioactive tracer molecule, PET-CT allows for the non-invasive imaging of (patho)physiological processes for the diagnosis of disease. Because of its inherently lower spatial resolution, PET-CT provides only limited anatomical detail but may provide valuable functional information complementary to that provided by modalities such as CT and MRI. Because functional disruptions frequently precede anatomical abnormalities, PET-CT is frequently able to detect abnormalities before conventional neuroimaging techniques.^2^

PET-CT has made a major impact on the management of several tumours; it also plays an important role in other clinical fields, including infectious disease, rheumatology, cardiology, and neurology. South Africa currently has 21 PET-CT cameras of which five (24%) are in the state sector (which manages at least 82% of the country’s patients),^3^ 13 (62%) are in the private sector (serving 18% of the country’s patients), and three (14%) are in dedicated research units (Table 1). Excluding research tracers that are compounded on site, these institutions have access to two commercially available radiotracers with neurological applications; these are fluorine-18 fluorodeoxyglucose (FDG), and fluorine-18 fluoro-dihydroxyphenylalanine (FDOPA).

**TABLE 1:** Provincial distribution of PET-CT scanners in South Africa.

Province	State	Private	Research
Eastern Cape	-	1	-
Free State	-	1	-
Gauteng	3	7	1
KwaZulu-Natal	1	3	-
Limpopo	-	-	-
Northern Cape	-	-	-
Western Cape	1	1	2

FDG is a radiolabelled analogue of glucose that is transported into cells via the same transporters before being trapped after conversion to FDG-6-phosphate.^4^ Since neurons are almost exclusively reliant on glucose to meet their energy requirements, FDG is a useful marker of regional neuronal activity, which is disrupted in many neurological conditions.^4^ FDOPA is a radiolabelled amino acid that is transported into presynaptic dopaminergic neurons via the large amino acid transporter before being converted to fluorodopamine which is then stored in presynaptic vesicles by the type-2 vesicular monoamine transporter.^5^

Uptake of FDOPA is a marker of presynaptic dopaminergic neuron integrity which is impacted early in the course of several degenerative movement disorders.^5^ Depending on their level of differentiation, both primary brain tumours and metastases exhibit variable upregulation of transporters for FDG and FDOPA.^6^

Neither of these tracers are associated with significant adverse effects, and the effective radiation dose from brain PET-CTs using FDG and FDOPA is low, typically in the order of 3.0 millisieverts (mSv) – 5.0 mSv, approximately equivalent to two CT brain studies.^4,5^

Proper patient preparation, standardisation of pre-scan conditions and attention to quality control are of paramount importance in brain PET-CT. Comprehensive recommendations for performing brain PET-CT studies have been published.^4,5^

While it is appropriate to investigate only a minority of neurological problems with PET-CT, for particular clinical indications, based on information available to the authors, PET-CT is underutilised in South Africa, especially in the private sector. Barriers to access may include the high cost of a brain PET-CTs (~R2000.00 – R12000.00) and PET tracers (~R6500.00 – R9000.00 per dose), the scarcity of PET-CT scanners, and the fact that many medical insurers do not cover these imaging studies under plan benefits. This is unfortunate, because in several specific scenarios, PET-CT can play an invaluable role in the correct diagnosis and hence management of many neurological conditions. Underutilisation may at least be in part attributable to lack of contemporary, evidence-based guidelines for the use of brain PET-CT in South Africa.

This article aims to briefly review the most common indications for PET-CT in neurological disorders in the South African context. While several applications for brain PET-CT were dealt with in the South African Appropriateness Guideline,^7^ this guideline was limited in its recommendations for neurological indications, and is now more than five years old – during which time additional evidence for the use of PET-CT in neurological conditions has accumulated. Strategies to improve access to PET-CT in the management of neurological conditions are discussed in this article.

## Methods

A topical approach was taken for this narrative review, which relied on pivotal papers known to the authors. Additional supporting meta-analyses, original research articles, reviews, and society endorsed practice guidelines were identified in targeted PubMed and Google Scholar searches using medical subject headings (MeSH) terms such as (‘positron-emission tomography’ OR ‘positron emission tomography computed tomography’) and (‘fluorodeoxyglucose F18’ OR ‘fluorodopa F18’) and the condition in question, for example, ‘dementia’, where relevant search results were further filtered by article type. Summaries were then prepared for the selected conditions.

### Ethical considerations

This article followed all ethical standards for research.

## Results

### Dementia

In 2015, it was estimated that 186 000 South Africans suffered from dementia^8^ which includes Alzheimer’s (AD), frontotemporal dementia (FTD), vascular dementia (VaD), dementia with Lewy Bodies (DLB), and dementia secondary to Parkinson’s disease (PD). HIV-associated dementia (HAD) is also relatively frequently encountered in the South African context, and is especially common in late stages of the disease. Exclusion of potentially surgically addressable causes of dementia justifies structural imaging with either CT or MRI (preferred), at least once in the workup of patients with cognitive impairment.^9^ PET-CT is, however, increasingly used as a complementary investigation, especially when structural imaging is normal. Professional society endorsed guidelines on the use of FDG PET-CT in neurodegenerative conditions have also recently been published.^10^

#### FDG PET-CT

**FIGURE 1:**
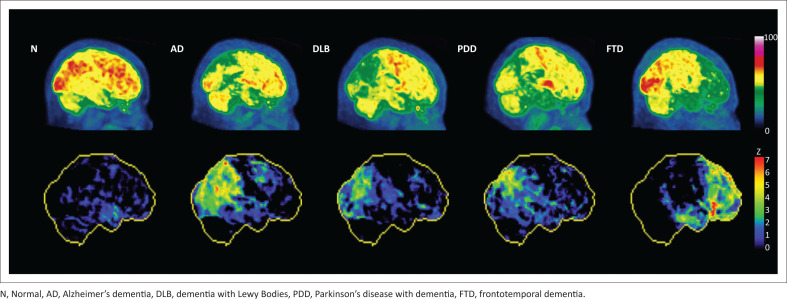
Fluorine-18 FDG PET scans in dementia. Maximum intensity projections of the PET scan (in the right lateral position) are shown on the top row (individually scaled for comparability). Corresponding statistical images (reductions compared to a normal database) are shown in the bottom row.

**FIGURE 2:**
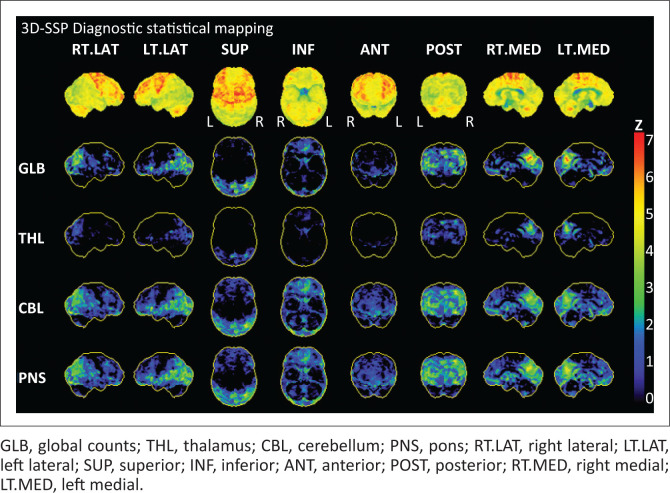
Example of a single-subject statistical analysis (decreases) for a FDG PET scan of a patient with dementia with Lewy Bodies. The top row represents surface projections of both brain hemispheres from different angles. Subsequent rows represent negative deviations from the normal database mean (z-score) for all brain regions, normalised to global counts (GLB), thalamus (THL), cerebellum (CBL), and pons (PNS). Similar outputs are generated for statistical *increases* (not shown). In this example, reduced metabolism in bilateral parietal occipital vortices is evident. Such analyses are highly recommended to support the interpretation of brain PETs.

In the context of *mild cognitive impairment*, FDG PET-CT may be valuable as a baseline investigation to diagnose early AD, FTD, or DLB, principally because of its high negative predictive value.^10^ In the context of *established dementia*, there is evidence to support the use of FDG PET-CT to diagnose AD when the clinical presentation is atypical, and to distinguish between the dementia subtypes (AD vs. FTD; DLB vs. AD; DLB vs. FTD; AD vs. VaD) when an alternate diagnosis is considered.^10^ Interpretation of FDG PET-CT scans performed to assist in diagnosis is aimed at identifying characteristic patterns of hypometabolism (as a result of neuronal degeneration) that assist in distinguishing dementia subtypes. For example, AD is predominantly a posterior dementia, with early involvement of the posterior cingulate cortex/precuneus region and adjacent temporoparietal association, progressing to greater frontal cortical involvement later, at which time characteristic sparing of sensorimotor and visual cortices may be evident.^10^ DLB is also a posterior dementia, but frequently spares the posterior cingulate (leading to the characteristic ‘cingulate island sign’) and may involve the primary visual cortex.^10^ Without clinical context, it may be impossible to distinguish the hypometabolic pattern in PD from AD or DLB.^10^ FTD, as the name implies, is associated with hypometabolism of the frontal lobes, with or without involvement of the temporal lobes, with spread to the parietal regions later in the course of the disease.^10^ The pattern of VaD is variable, depending on the location, severity, and extent of ischaemia. PET with FDG currently plays only a limited clinical role in the diagnosis of HAD, though the test may be beneficial in older patients, to distinguish the condition from other dementing diseases. Examples of FDG PET scans in different dementia subtypes are illustrated in Figure 1. Whether FDG PET-CT is performed for mild cognitive impairment (suspected dementia) or in established dementia, single-subject statistical analysis should be performed to support nuclear physician interpretation.^10^ During such analyses, a patient’s PET scan is re-oriented, normalised for intensity, and then spatially warped to fit a standard template, before it is compared to an age-appropriate database of normal brain scans in order to generate maps of statistical deviation from the mean (Figure 2).

#### FDOPA PET-CT

FDOPA PET-CT is a sensitive and specific tool to evaluate the integrity of nigrostriatal dopaminergic structures.^5^ In addition to its main role in the selective evaluation of patients with movement disorders (see the following section), it may be clinically valuable to distinguish between AD and DLB (PD and DLB) in difficult cases,^11^ occasionally in combination with the results of FDG PET-CT. The distinction between these two clinical entities is important, since patients with DLB have heightened sensitivity to neuroleptics, occasionally with lethal consequences.^11^

### Movement disorders

The crude prevalence of idiopathic PD in sub-Saharan Africa varies between countries, from 7 to 20 per 100 000.^12^ The prevalence of other Parkinsonian disorders and essential tremor is unknown.

The diagnosis of PD is primarily a clinical one, with neuroimaging typically reserved as ancillary, when there are atypical clinical features.^13^ In such cases, transcranial sonography, MRI, and nuclear techniques such as single photon emission computed tomography (SPECT) and PET may all play a role.^13^ PET-CT is most useful in confirming dopaminergic degeneration and to a limited degree in differentiating between PD and PD mimics, especially when invasive intervention in considered. Importantly, PET-CT does not currently play a clinical role in non-Parkinsonian movement disorders such as dystonia, hereditary spastic paraplegias, or paroxysmal dyskinesias.

#### FDOPA PET-CT

**FIGURE 3:**
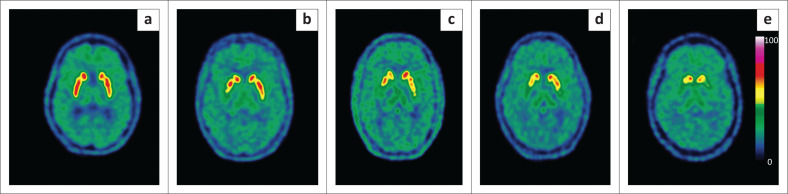
Fluorine-18 FDOPA PET scans in five patients with movement disorders demonstrating absence of dopaminergic deficit (**a**) in a patient with essential tremor, and progressive stages of dopaminergic deficit (**b – e**) in patients with neurodegenerative disorders of the striatum.

FDOPA is an analogue of dihydroxyphenylalanine – an amino acid precursor used in the synthesis of endogenous dopamine. As such, it has emerged as a sensitive biomarker of presynaptic dopaminergic degeneration in the caudate and putamen. Evidence suggests that FDOPA PET-CT can be substituted for Iodine-123-N-fluoropropyl-2-beta-carbomethoxy-3-(4-iodophenyl)nortropane (I-123 FP-CIT) or I-123 beta-CIT SPECT to evaluate presynaptic dopaminergic integrity.^5^ When patients present with typical features of PD and respond to levodopa therapy, there is usually no need for nuclear neuroimaging. However, even in expert hands, approximately 25% of the patients with PD are misdiagnosed on clinical grounds,^14^ and thus if the clinical phenotype is atypical, or there is little or no response to levodopa, FDOPA PET-CT can be of benefit to confirm or exclude the presence of dopaminergic degeneration, with a high sensitivity and specificity.^5^ This is true of distinguishing *non-degenerative* Parkinsonian mimics such as psychogenic, vascular, and drug-induced Parkinsonism and essential tremor from *degenerative* dopaminergic disorders, which include PD, corticobasal degeneration (CBD), progressive supranuclear palsy (PSP) and multisystem atrophy (MSA).^5^ In degenerative conditions, such as PD, there is progressive loss first of putaminal and then caudate uptake. Frequently these changes are asymmetrical, typically worse on the side contralateral to the most affected limb.^5^ Examples of FDOPA scans in patients investigated for movement disorders are illustrated in Figure 3.

**FIGURE 4:**
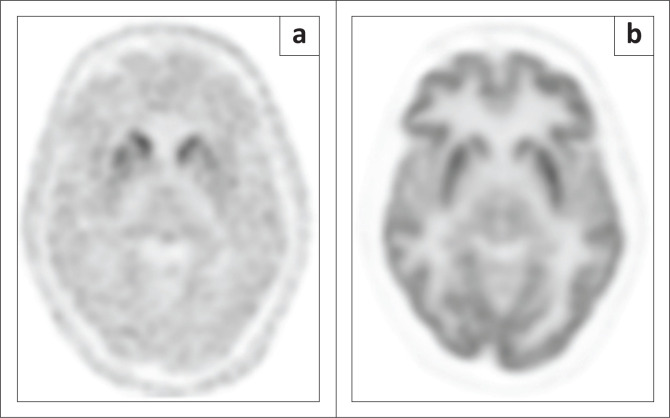
Fluorine-18 FDOPA (a) and FDG (b) PET scans in a patient with a movement disorder. The FDOPA PET convincingly demonstrates dopaminergic loss in bilateral putamina, while the FDG study demonstrates normal metabolism. These features are consistent with a diagnosis of idiopathic Parkinson’s disease.

It should be noted that FDOPA PET-CT, like all presynaptic dopaminergic imaging techniques, is unable to distinguish between the different degenerative dopaminergic disorders. Evidence suggests that in the event that this is potentially of clinical importance, FDG PET-CT performs better than post-synaptic dopaminergic imaging,^15^ tracers for which are in any case not currently available in South Africa. Example scans of a patient investigated for a movement disorder with FDOPA and FDG PET are provided in Figure 4.

#### FDG PET-CT

Dopaminergic degenerative conditions other than PD, the so-called ‘Parkinson-Plus’ syndromes are difficult to treat and have a poor prognosis. There is evidence-based guideline endorsement for the use of FDG PET-CT to distinguish PD from PSP^10^ and to support the diagnosis of CBD.^10^ Characteristic reduction in metabolism on FDG PET-CT is one of the diagnostic criteria for possible MSA.^16^ The FDG PET-CT features of the different Parkinsonian syndromes are complex, and beyond the scope of this review.^17^

### Epilepsy

The prevalence of epilepsy in South Africa is unknown, but is likely high. Approximately one-third of the patients with epilepsy suffer from seizures that are refractory to medication^18^ and may be suitable for surgical management.

#### FDG PET-CT

**FIGURE 5:**
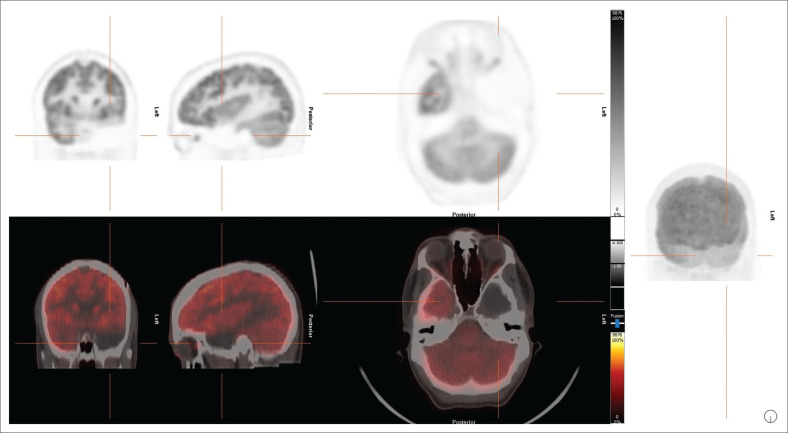
Interictal FDG PET scan in a patient with intractable temporal lobe epilepsy being worked up for epilepsy surgery. The scan demonstrates marked hypometabolism in the left temporal lobe, confirming lateralisation of the epileptogenic focus. The right panel demonstrates the PET maximum intensity projection; top row - PET orthogonal slices; bottom row - fused (PET-CT) orthogonal slices.

MRI is the imaging modality of choice in identifying potentially resectable epileptogenic lesions, but will be negative or inconclusive in up to half of the patients.^19^ Interictal PET-CT with FDG is a useful complementary tool in the workup of such patients, in whom the causal lesion is typically associated with a focal region of hypometabolism.^19^ Evidence suggests that FDG PET-CT is able to lateralise the epileptogenic origin (Figure 5) in more than 80% of temporal lobe epilepsy cases and while less sensitive in extra-temporal lobe variants, still plays an important role in guiding subdural electrode placement in invasive stereo-electroencephalography and is highly prognostic of seizure-free status post-surgery.^19^ Best results using PET-CT are achieved with a combination of expert visual analysis and statistical methods with specialised neuro-analysis software tools.^20^ It is important, when performing PET-CT for this indication, that patients are correctly prepared and that interictal status is confirmed prior to radiopharmaceutical injection.^4^

### Brain tumours

Brain tumour epidemiology in South Africa is uncertain. The high prevalence of HIV in South Africa likely influences primary brain tumour incidence, since there is evidence that meningiomas occur at a younger age, and glioblastomas occur at a greater frequency in HIV-positive individuals.^21^ Certainly, primary central nervous system lymphoma (PCNSL) in South Africa is mostly seen in the context of HIV, in which group it accounts for up to 15% of Non-Hodgkin’s lymphomas.^22^ Brain metastases are known to develop in almost 30% of patients with solid tumours.^23^ There are many different brain tumours, but the clinical role for PET-CT is confined principally to three broad pathological subtypes which are considered here, namely gliomas, PCNSL, and brain metastases.

Management of most gliomas relies on a multidisciplinary combination of surgery, radiotherapy and chemotherapy.^24^ Primary central nervous system lymphoma is managed with chemotherapy and, in HIV-positive patients, the addition of antiretroviral therapy.^22^ Surgery or radiosurgery and/or radiotherapy are often considered to locally address brain metastases since many systemic cytotoxic medications fail to penetrate the blood-brain barrier.^23^ Several second-line options are available to treat both recurrent gliomas and recurrent metastases, making the identification of recurrence important. MRI is the primary modality for assessing primary and secondary brain tumours at all disease stages. Assessing treatment response and identifying recurrence of brain tumours may however prove challenging on MRI, because of the effects of tumour oedema and radiation necrosis post-therapy.^25^

#### FDOPA PET-CT

**FIGURE 6:**
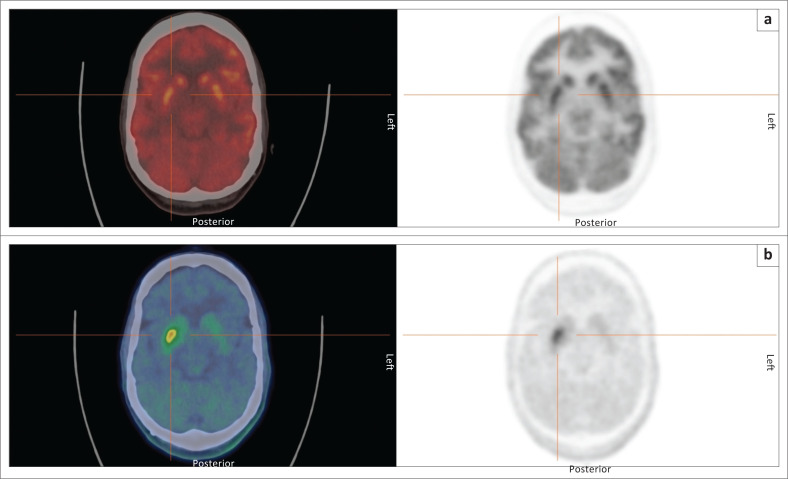
Fluorine-18 FDG (a) and FDOPA (b) PET-CT (fusion) and PET images of a patient with suspected recurrence of a low-grade glioma. The FDG PET-CT study was normal, while FDOPA PET-CT (preferred) demonstrates clear recurrence in the right putamen. This recurrent lesion was initially missed on MRI.

In contrast to FDG, the physiological uptake of FDOPA in normal brain tissue is (except for in the striatum) low, which makes even subtle increases in uptake appreciable. In addition, increased FDOPA uptake in inflammatory processes appears to be less of an issue compared to that seen with FDG. Increased signal on FDOPA PET-CT is thus a sensitive and specific marker for pathologies with upregulated expression large amino-acid transporters 1 and 2 (LAT1 and LAT2), which include gliomas and cancers that metastasise to brain.^6^ Evidence supports the use of FDOPA PET-CT in gliomas in evaluating tumour extent at baseline for the purpose of surgery and radiotherapy planning, and in evaluating treatment response in patients treated with antiangiogenic therapy (bevacizumab),^26^ as well as in identifying tumour recurrence post-therapy in both gliomas and brain metastases.^6,26^ There is currently insufficient evidence to support the use of FDOPA PET-CT in distinguishing between neoplastic and non-neoplastic tissue or in establishing tumour grade. An example of FDOPA PET in the evaluation of recurrent glioma is provided in Figure 6.

#### FDG PET-CT

For the assessment of recurrence in all glial tumours, and in brain metastases, FDOPA PET-CT is preferred over FDG PET-CT when MRI is equivocal.^6,26^ However, when FDOPA is not available, localised increase in FDG uptake may be useful in identifying tumour recurrence in patients with anaplastic glial and glioblastoma histologies,^24^ and in patients with brain metastases with high FDG avidity.^27^ Evidence suggests that when used to detect recurrence of low-grade gliomas, FDG PET-CT is less sensitive,^28^ and by a similar rationale, FDG PET-CT should not be used to detect recurrence of treated brain metastases when these occur in the setting of a primary tumour with low FDG avidity. Neither should FDG PET-CT be used for the primary diagnosis nor for grading of gliomas. Whole-body FDG PET-CT is occasionally used in the workup of an unknown primary in a patient with brain metastases.^24^

Although FDG PET-CT may play a role in differentiating PCNSL from toxoplasmosis in HIV-positive patients (avid uptake is described in PCSNL vs. typically minimal to absent uptake in toxoplasmosis), the evidence for this indication is limited.^29^ In the context of confirmed PCNSL, the performance of whole body PET-CT is appropriate to exclude the presence of synchronous systemic lymphoma, and to evaluate suspected systemic progression.^24^

### Neuropsychiatric lupus and immune-mediated encephalitides

The incidence and prevalence of lupus in South Africa are unknown, but it is likely that the disease is common, and frequently severe. Throughout the world, paraneoplastic and autoimmune encephalitis are rare but represent important, treatable conditions.

Brain involvement in systemic lupus erythematosus (SLE) is not unusual, and represents a serious but treatable complication.^30^ Up to 80% of SLE patients will develop psychiatric or neurological symptoms.^31^ In about 40% of such cases, symptoms occur by secondary mechanisms (e.g. related to infection or systemic organ dysfunction), with the remaining 60% being attributable to direct disease involvement of the brain.^32^ The diagnosis of neuropsychiatric-SLE (NPSLE) is difficult and should be based on both the clinical presentation as well as the results of special investigations, which may include lumbar puncture; nerve conduction tests (for peripheral neuropathies); and electroencephalography.^30^ In terms of neuroimaging, MRI is the preferred first-line imaging method, with nuclear medicine imaging techniques such as perfusion SPECT and FDG PET considered as ancillary.^30^ Part of the difficulty in diagnosing NPSLE is that its pathophysiology is very likely multifactorial, related to autoantibodies, pro-inflammatory cytokines, microangiopathy and vasculitis.^30^

Similarly, PET-CT may play a complementary role to MRI in antibody-mediated encephalitides, whether these occur because of antibodies to intracellular neuronal proteins (paraneoplastic encephalitis syndromes), or due to antibodies targeting neuronal cell membrane/synaptic proteins (autoimmune encephalitis syndromes).^33^

#### FDG PET-CT

**FIGURE 7:**
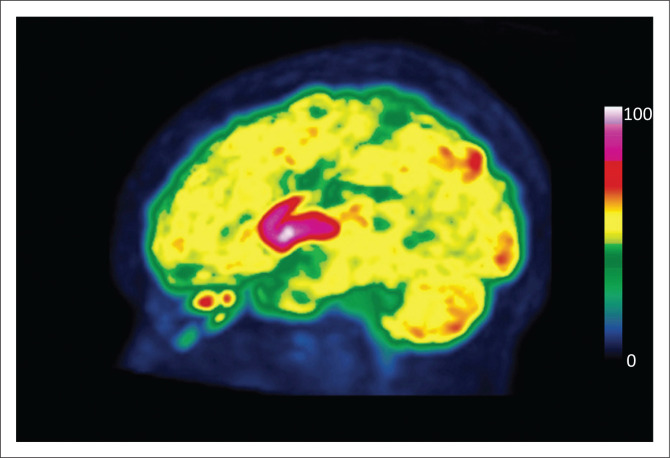
Fluorine-18 FDG PET (maximum intensity projection) in a patient known with systemic lupus erythematosus who presented with new-onset of choreoathetoid movements. Intense, symmetrical uptake in the striatal nuclei is consistent with a known neurological manifestation of systemic lupus erythematosus. MRI in this case reported non-specific features of microangiopathy.

FDG PET-CT may be considered to assist in the diagnosis of NPSLE when the clinical picture is strongly suggestive, but MRI is normal or doubtful.^30^ One study identified abnormalities on FDG PET-CT in 75% of such cases.^34^ Parieto-occipital hypometabolism is most frequently described, though hypometabolism in other regions and occasional hypermetabolic lesions may also be observed.^30^ Increased uptake in the striatal nuclei has been described in patients with antiphospholipid syndrome and chorea (Figure 7).^35^

In autoimmune encephalitis, FDG PET-CT of the brain may play an important role in establishing the diagnosis, especially when MRI is negative or equivocal, and in antibody negative cases.^36^ Often, characteristic hypermetabolism is seen in the limbic regions in these patients, although abnormalities in the brainstem, cerebellum and cerebral cortex are also described.^36^ In paraneoplastic encephalitis, whole body FDG PET-CT may be warranted in identifying the site of the primary tumour depending on the clinical syndrome, the type of antibodies present and the results of conventional anatomical imaging.^33^

### Brain infections

Africa has the world’s highest incidence of central nervous system (CNS) infections in the region of 770/100 000 per year.^37^ The worldwide incidence of opportunistic CNS infections in HIV-positive patients has fallen drastically since the widespread rollout of antiretroviral therapy but remains a problem in Africa, including South Africa.

PET-CT plays a minimal role in brain infections but is occasionally useful when structural imaging is inconclusive. Limited evidence suggests possible roles for FDG PET-CT to distinguish between toxoplasmosis and PCNSL in HIV-positive patients,^29^ and as an occasional adjunct in the diagnosis of encephalitis.^38^ Research demonstrating a clear benefit of FDG PET-CT in patients with infective meningitis is lacking. There are currently no recognised clinical indications for FDOPA PET-CT in the management of brain infections.

### Traumatic brain injury

South Africa has high rates of interpersonal violence and road traffic accidents. While there are no figures on the epidemiology of either severe or mild traumatic brain injury (TBI), the contribution of TBI to morbidity and mortality is likely substantial.

Regional uptake of FDG is frequently reduced after TBI, even when the injury is mild^39^ making it an attractive research tool in the study of this condition. There is however insufficient evidence to support the use of FDG PET-CT in TBI for clinical or medicolegal purposes. Nuclear physicians should always enquire about previous history of head injury when interpreting FDG PET-CTs performed for other indications, to avoid this potential confound.

### Psychiatric and neurodevelopmental disorders

With the possible exception of using FDG PET-CT to distinguish depressive pseudo-dementia (anecdotally supported by an expert panel),^10^ PET-CT currently plays no role in the clinical management of these disorders on a single-subject level.

## Discussion

There is evidence that brain PET-CT with FDOPA or FDG is frequently clinically invaluable. South Africa is in many respects fortunate to have an established PET-CT infrastructure and access to these two PET tracers which have well-defined applications in neurological disorders. Despite this and the opportunity to improve patient care, PET-CT likely remains underutilised.

This article has reviewed the most common clinical applications for brain PET-CT. It is important to emphasise that indications exist outside the scope of this review for which PET-CT may be appropriate, and that these indications evolve with emerging evidence and as new tracers become available. There is a need for evidence-based South African guidelines to assist clinicians, nuclear medicine physicians and medical insurers. In addition, it is incumbent on clinicians to incorporate brain PET-CT into professional societal guidelines when there is sufficient evidence to support doing so.

Several barriers exist to accessing PET-CT scans, which must be overcome. One of these is the scarcity of PET-CT scanners in some regions. This is most apparent in the state sector and will only be addressed through high-level strategic planning by provincial health departments engaging with nuclear physicians and clinicians. It is important that as part of such consultations, doctors provide the necessary evidence supporting the use of PET-CT as well as (ideally) cost-effectiveness data. Given that most PET-CT work is performed for oncological indications, such data would necessarily need to focus predominantly on PET-CT’s utility and its cost-effectiveness in cancer management. Related to this, prices for brain PET-CTs may frequently be prohibitive and there is a need to explore strategies that might reduce costs and so improve patient access.

In the authors’ experience, many South African nuclear physicians believe they lack the expertise and/or confidence to report these studies. There are several possible strategies to address this. Firstly, nuclear medicine practitioners should familiarise themselves with the main guidelines.^4,5,10,26^ Brain PET-CT requires careful attention to study indication, patient preparation, acquisition and quality control, and interpretation as detailed in these documents. Secondly, interpretation of brain PET-CTs should be limited to nuclear physicians with adequate training and expertise in this field, and with access to the necessary quantitative tools. Thirdly, nuclear physicians should arrange additional training in the performance and interpretation of nuclear neuroimaging by petitioning their professional societies for workshops or other educational forums in which their requirements are addressed. Finally, during interpretation of brain PET-CTs, the use of semiquantitative statistical scoring tools enhance specificity, accuracy and reader confidence, and are especially valuable for less experienced readers.^40^

The main strength of PET-CT is its ability to image biological processes, which are typically disturbed before appreciable anatomical changes occur in disease.^2^ The main limitations of PET are its lower spatial resolution when compared to MRI and CT, and its limited specificity, especially with respect to FDG PET. These weaknesses are circumvented when PET is used in combination with structural techniques, in the appropriate clinical context. Nuclear neuroimaging techniques such as PET-CT should in other words be considered as complementary to MRI and CT. In such an ancillary capacity, brain PET-CT has several evidence-based applications in the fields of gerontology, neurology, neuro-oncology, rheumatology and infectious disease. The value of multidisciplinary team management is now widely accepted in clinical medicine and in the authors’ opinion there is a growing rationale to include nuclear physicians as partners in improving patient care. This will become even more important as neurological radiotracers already available internationally become available in South Africa in the future.

## Conclusion

Brain PET-CT with FDG or FDOPA may be currently underutilised in South Africa. Nuclear neuroimaging techniques have several evidence-based applications which may warrant greater use in clinical practice. There is a need for evidence-based South African guidelines to assist clinicians, nuclear medicine physicians and medical insurers in selecting patients appropriately imaged with PET-CT.
